# Migrants in Greece and mental health issues

**DOI:** 10.1192/j.eurpsy.2022.1406

**Published:** 2022-09-01

**Authors:** A. Zartaloudi

**Affiliations:** University of West Attica, Nursing, Athens, Greece

**Keywords:** Greece, mental health, migration

## Abstract

**Introduction:**

Migration is a difficult and painful process for individuals, since they could no longer rely on the supportive structures of their own country that would help them develop resilience and mental well-being, on the one hand and, on the other hand, they may be obliged to find a new identity and adapt to a new social context.

**Objectives:**

To identify mental health issues in migrants in Greece.

**Methods:**

A literature review has been made through PubMed database.

**Results:**

First-generation immigrants exhibited an increased risk of poor mental health including increased levels of depression, post-traumatic disorder and anxiety compared to local population. When immigrants come to a new country, they often experience culture shock, significantly influencing their mental health. The term “culture shock” describes feelings of weakness and a state of disorientation of individuals living in a new environment as well as the difficulties they face in the process of their adapting to the new conditions. Individuals lack a social supportive environment while experiencing lack of acceptance, as well as social discrimination, economic exploitation and racism by local society. Additionally, their cultural background can influence and differentiate the way they perceive, react and cope stressful conditions.

**Conclusions:**

For the smooth completion of the cultural process, mutual adaption to the new conditions of both migrants and host society is needed, focusing on the understanding of different cultural heritage, as well as on the respect and recognition of rights of both sides.

**Disclosure:**

No significant relationships.

